# Self-Amplifying RNA Approach for Protein Replacement Therapy

**DOI:** 10.3390/ijms232112884

**Published:** 2022-10-25

**Authors:** Dimitri Papukashvili, Nino Rcheulishvili, Cong Liu, Yang Ji, Yunjiao He, Peng George Wang

**Affiliations:** Department of Pharmacology, School of Medicine, Southern University of Science and Technology, Shenzhen 518000, China

**Keywords:** saRNA, protein replacement, protein deficiency, single-gene disorders, alpha-1 antitrypsin deficiency, AATD, mRNA, taRNA

## Abstract

Messenger RNA (mRNA) technology has already been successfully tested preclinically and there are ongoing clinical trials for protein replacement purposes; however, more effort has been put into the development of prevention strategies against infectious diseases. Apparently, mRNA vaccine approval against coronavirus disease 2019 (COVID-19) is a landmark for opening new opportunities for managing diverse health disorders based on this approach. Indeed, apart from infectious diseases, it has also been widely tested in numerous directions including cancer prevention and the treatment of inherited disorders. Interestingly, self-amplifying RNA (saRNA)-based technology is believed to display more developed RNA therapy compared with conventional mRNA technique in terms of its lower dosage requirements, relatively fewer side effects, and possessing long-lasting effects. Nevertheless, some challenges still exist that need to be overcome in order to achieve saRNA-based drug approval in clinics. Hence, the current review discusses the feasibility of saRNA utility for protein replacement therapy on various health disorders including rare hereditary diseases and also provides a detailed overview of saRNA advantages, its molecular structure, mechanism of action, and relevant delivery platforms.

## 1. Introduction

Despite significant progress in science and medicine, until now, there are a number of diseases for which the development and improvement of new strategies are emerging. The potential of messenger RNA (mRNA) application is enormous for the well-being of humans [1]. Compared with the traditional vaccine, making the mRNA vaccine has numerous advantages. In the case of mRNA vaccine administration, the human body makes proteins itself, and huge bioreactors are no longer needed for vaccine production. Therefore, the human body becomes a bioreactor itself, and the time for making a vaccine is markedly reduced [2,3]. There are two types of mRNA, conventional–non-replicating RNA and self-replicating, in other words, self-amplifying RNA (saRNA). saRNA has many structural similarities to conventional mRNA: it is a linear, single-stranded RNA molecule that is synthesized with a 5′ cap, 3′ polyA tail, and 5′ and 3′ untranslated regions (UTRs). The main structural difference is that saRNA is a much larger molecule as it encodes four extra proteins in addition to the protein of interest or vaccine antigen. These non-structural proteins (nsPs) that encode a replicase are usually derived from an alphavirus [4]. saRNA vaccine is proposed to have features of an updated version of the conventional mRNA vaccine with multiple advantages, especially its low dosage [5], reduced side effects [4], and long-lasting outcomes [6]. It is shown that 64-fold less material is needed in case of saRNA vaccine development compared with the conventional mRNA to achieve the same result for producing influenza virus antigens [6]. Hence, it also makes the approach less costly. Meanwhile, mRNA technology is also preclinically tested effectively for protein replacement therapy in certain health complications such as heart disease [7] and alpha-1 antitrypsin deficiency (AATD) [8]. In addition, it is hypothesized that saRNA can be used for preventing Alzheimer’s disease (AD) by raising adenosine triphosphate (ATP) levels in the brain via reducing amyloid beta (Aβ), pyroglutamate-modified Aβ, and cyclophilin-D [9]. saRNA has great potential to open a new platform in medicine to produce a drug in simple and inexpensive ways. Therefore, it might alleviate the process and save millions of lives that are suffering not only from infectious diseases but also from hereditary or metabolic disorders as well. In case of infectious diseases, for fighting the coronavirus disease 2019 (COVID-19) pandemic, which is caused by severe acute respiratory syndrome coronavirus 2 (SARS-CoV-2), the use of the saRNA vaccine has also been suggested [10]. Indeed, saRNA vaccine use has already been widely assessed against COVID-19, and there are a number of preclinical [11] and ongoing clinical studies. Remarkably, Arcturus Therapeutics is working on saRNA vaccine development for COVID-19, and the current stage of clinical development is Phase 3 in Vietnam (NCT05012943) [12,13]. saRNA strategy seems to be promising for protein replacement in certain diseases. This approach should also be considered for cosmetic uses such as alopecia treatment. In case of androgenetic alopecia (AGA), it has been proposed that microRNAs (miRNAs) have the ability to inhibit certain proteins to activate signaling pathways involved in this process. On the other side, the saRNA approach also has a huge potential to activate certain signaling pathways implicated in the hair growth process (such as Wnt/β-catenin) by an augmentation of certain peptides (Wnt ligand, β-catenin, etc.) into dermal papilla cells and initiate hair growth [14].

Apparently, the mRNA vaccine approach has multiple benefits compared with the conventional way of vaccine development [2,15]. Indeed, the saRNA approach allows us to use the advantage to produce even more proteins inside the body when it is needed for a longer period of time compared with the conventional mRNA technique. Therefore, the saRNA approach appears to have promising outcomes compared with the other techniques of vaccines and therapies. However, despite the auspicious results of the mRNA approach, more preclinical and clinical studies are warranted on mRNA and saRNA-based therapies for protein replacement. Therefore, here, the need for the initiation of studies directed at protein replacement therapy based on accurate designing and administration in precise doses of saRNA constructs is first proposed. This review summarizes the recent and relevant studies on protein replacement therapy using saRNA technology. Moreover, in order to facilitate this direction and solve numerous problems that currently exist, considering treating various health disorders including the rare congenital diseases saRNA approach seems to be the right target. In addition, the mechanism of action of saRNA, as well as current challenges and limitations of this approach, are also reviewed.

## 2. Clinical Trials of mRNA Approach for Protein Replacement Therapy

saRNA technology indeed represents a promising approach for advancing the field of vaccinology and protein replacement therapy for various diseases. The manufacturing process of the vaccine based on saRNA technology is entirely synthetic. It does not require a viral culture or any complicated process that is usually related to the manufacturing process of conventional licensed vaccines [16]. Basically, all the existing evidence about mRNA vaccine use can be substituted with an saRNA approach that might even overcome certain difficulties of conventional mRNA application [17]. Indeed, saRNA is currently in clinical development for SARS-CoV-2 intensively (the trials are registered as NCT05012943; NCT05037097; ISRCTN17072692, NCT04758962, EudraCT 2020-001646-20). Apart from clinical trials, there are a number of saRNA approaches that undergo preclinical testing for various infectious diseases such as influenza, rabies, etc. Besides the infectious diseases, it can also be used as a powerful tool for cancer treatment as there are studies on the mRNA vaccine approach for cancer therapy [17,18,19,20,21]. Using the mRNA vaccine for cancer treatment is a new approach compared to its initial use, which is the prevention of infectious diseases [22]. Undoubtedly, there will be obstacles that need to be overcome related to saRNA use for cancer immunotherapy. However, unlike conventional mRNA vaccines, this way of treatment has greater potential with its self-amplifying function, hence it may facilitate the developing process for generating the finest mRNA-based therapy against cancer. Interestingly, there are a number of studies in clinical trials using the mRNA approach for protein replacement for the treatment of certain diseases (Table 1).

## 3. Major Differences between saRNA & mRNA Technologies

**Figure 1:**
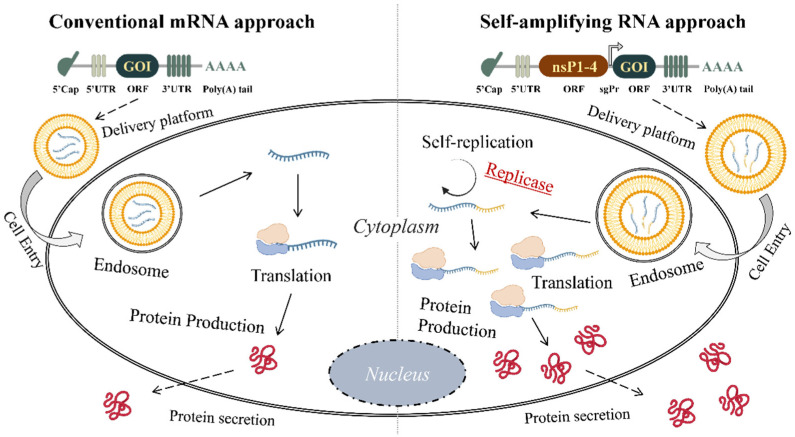
Protein production via mRNA and saRNA approaches. UTR, untranslated region; GOI, gene of interest; ORF, open reading frame; nsP1-4, non-structural protein 1-4; sgPr, subgenomic promoter.

While plasmid DNA has advantages compared to direct protein therapy for protein substitution, mRNA is superior to the two strategies mentioned [23,24]. Most importantly, mRNA vaccine therapy saves a lot of time, which is equal to saving millions of lives. Compared with traditional ways of vaccine preparation, which require big bioreactors, in case of the mRNA vaccine approach, antigens of interest are autologously produced by the cell’s machinery itself [2]. The advantages of mRNA are conditioned by several facts. First of all, mRNA, which is delivered into the host cell, does not need to be transported into the nucleus as happens in the case of DNA. It is translated into protein in the cytoplasm [25] that does not alter the physiological state of the cell. This averts any possible intervention in the human genome and makes it a safe and feasible strategy [26]. On the other hand, saRNA provides advancement for protein replacement [27]. The main differences between conventional mRNA and saRNA vaccine technologies are as follows: in case of saRNA, a lower dose of vaccine is required compared with the conventional mRNA-based immunization strategy. This characteristic is conditioned by the fact that only a small amount of saRNA is necessary for producing large amounts of antigens in the body. For example, Vogel et al. assessed the difference between the effects of mRNA and saRNA vaccines against influenza virus in BALB/c mice and revealed the protective efficiency of both approaches; however, the same level of protection was observed via 80 mg mRNA and 1.25 mg saRNA [6]. Furthermore, if comparing the doses of the currently available two SARS-CoV-2 mRNA vaccines by Moderna (mRNA-1273) and BioNTech/Pfizer (BNT162b2) that are 100 µg and 30 µg, respectively [2], in a clinical trial (Phase 3) on saRNA vaccine (ARCT-154) against COVID-19 by Arcturus Therapeutics, only 5 µg saRNA are used (NCT05012943). In addition, the saRNA vaccine has long-lasting efficiency, comparatively fewer side effects, and is more cost-effective per dose. The length of the two types of RNA also differs—saRNA construct is longer (~10,000 nt) unlike mRNA (~2000 nt) [28]. According to the abovementioned, the application of saRNA approach seems to be rational not only for vaccine development against infections but also for protein replacement therapy (Figure 1).

## 4. saRNA—Mechanism of Action

**Figure 2:**
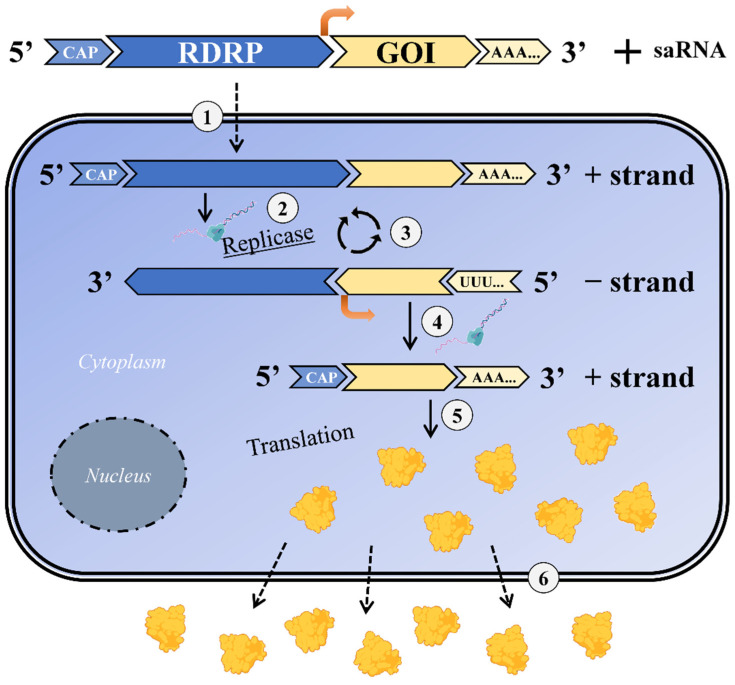
saRNA mechanism of action. 1. saRNA enters the cells; 2. Replicase is translated which uses saRNA as a template to make a complementary negative saRNA (-saRNA) strand; 3. Self-amplification takes place: replicase also uses this -saRNA as a template to make more positive saRNA (+saRNA); 4. In addition, replicase can recognize the sgPr in the negative strand from which a sgRNA of positive polarity (+sgRNA) is synthesized; 5. sgRNA is then translated into desired antigen or protein at very high levels; 6. Protein is released from the cell. RDRP, RNA-dependent RNA polymerase; GOI, gene of interest; saRNA, self-amplifying RNA.

The saRNA vector is a positive-strand RNA (+saRNA) containing the genes coding for the viral replicase and the gene of interest (GOI) downstream of a subgenomic promoter (sgPr). The replicon is based on the alphavirus nsPs. There are three types of alpha-viruses that are used in the saRNA approach: Venezuelan Equine Encephalitis Virus—VEEV (commonly used), Semliki Forest Virus—SFV, and Sindbis Virus. By deleting the viral structural proteins, the RNA cannot produce an infectious virus. After being delivered to the cytoplasm, the nsPs form an RNA-dependent RNA polymerase (RDRP). Each of the nsPs plays a role in the formation of the RDRP: nsP1 is required for 5′ capping of viral RNA. nsP2 has helicase and protease activity. On the one hand, it unwinds the RNA duplex during replication, while, on the other hand, it cleaves the polyprotein into individual nsPs [29]. nsP3 plays an essential role in the interactions between viral and host proteins [30]. nsP4 is an RDRP and is the first nsP which is cleaved from the polyprotein [29]. As saRNA contains the replicon of alphaviruses’ nsPs, they can be self-amplified in the host cell; thus, a huge number of desired proteins are produced [31,32]. RDRP replicates both the entire RNA strand and sgRNA [32]. This RNA replication leads to a higher and long-lasting antigen expression compared to the non-replicating mRNA [9,33]. The mechanism of how saRNA works after the delivery is based on the following: saRNA enters the cells where the replicase can be directly translated, being able to use saRNA as a template to make a complementary negative saRNA (−saRNA) strand. Replicase can also use this −saRNA as a template to make more +saRNA, allowing its self-amplification. On the other hand, replicase can recognize the sgPr in the negative strand from which a subgenomic mRNA (+sgRNA) of positive polarity is synthesized. sgRNA can be translated to produce the desired protein at very high levels, which will be secreted if having a corresponding signal peptide [31,34,35]. The schematic illustration is given in Figure 2.

After the delivery to the host cells, saRNA as well as the double-stranded RNA intermediates produced during the self-replication [36] stimulate an innate immune system as they are recognized as non-self-molecules [37]. This recognition leads to the secretion of interferon (IFN) leading to impeding the successful translation of saRNA. First, pattern recognition receptors (PRRs) are stimulated. According to the cellular location, saRNA can activate the retinoic acid-inducible gene I (RIG-I), melanoma differentiation-associated protein 5 (MDA5), protein kinase R (PKR), 2′-5′-oligoadenylate synthetase (OAS), and possibly activate other pathways too that are present in the cytosol [3]. On the other hand, via endosomal sensing, saRNA and its replication intermediates can induce the activation of toll-like receptors (TLRs) which are present in the endosomes [3]. The saRNA sensing leads to the production of type I IFN and other cytokines [38] that ultimately induce the maturation of dendritic cells (DCs), activation of T helper cells, and T cell-dependent B cells [37]. For escaping cytosolic and endosomal sensing of in vitro transcribed mRNA and, thus, avoiding the undesirable innate immune response, nucleoside modification can be performed as developed by Kariko and coworkers [39]. However, the same cannot be completed in case of saRNA as its replication is based on the host-cell factors, making the addition of modified nucleosides not reasonable as these modifications would vanish after the first round of amplification. To overcome this obstacle, Blakney et al. have proposed the potential strategy of diminishing the outcome of type I IFN activation via including the ORF of innate inhibiting proteins (IIPs) directly in saRNA construct. For that, they have used the same mechanism that allows the RNA viruses escape innate immune recognition. The library of saRNA constructs encoding IIPs was screened, and the study demonstrated that IIPs enhance the saRNA expression [40].

## 5. Potential Application of saRNA for Non-Infectious Health Disorders

In terms of infectious diseases, the COVID-19 outbreak already showed us how important preparedness is for future pandemics, therefore, along with the conventional mRNA approach, saRNA indeed represents one of the main targets for quick design and development of vaccines for any outbreaks in the future. It is already evidenced that the mRNA vaccine works effectively against the COVID-19 pandemic; hence, research on advancing the mRNA approach has also started. There are a number of clinical trials based on mRNA technology for protein replacement. However, it would be more rational to use the RNA technology for protein replacement, which would result in the production of more proteins with a lower dose while eliciting a long-lasting effect. More importantly, the administration of a lower dose is associated with reduced side effects and the approach itself is cost-effective. However, it is also noteworthy that there is no saRNA-based drug approved in clinics so far, although the promising results are demonstrated by Arcturus Therapeutics and they currently are in clinical trial Phase 3 for saRNA vaccines against COVID-19. Currently, most of the scientific attention is paid to infectious diseases, while there are a number of other diseases that deserve a proper fight and should not be forgotten. Since the start of the current pandemic, fighting infectious diseases became the main target for researchers and more efforts are put in place against SARS-CoV-2 and other infectious diseases (Table 2), while other health disorders should not be forgotten as well.

Therapeutic in vitro transcribed-saRNA can be used for encoding the downregulated proteins that are responsible for diseases. These genomic defects and other diseases include Fabry disease, which is associated with the deficiency of alpha-galactosidase A, propionic acidemia, which is manifested with the deficiency of an enzyme propionyl-CoA carboxylase, hemophilia B, which is a condition of blood coagulation defects and is caused by the deficit of coagulation factor IX, ornithine transcarbamylase (OTC) deficiency, cystic fibrosis caused by the defect of cystic fibrosis transmembrane conductance regulator, diabetes mellitus (DM) caused by the deficiency of insulin, etc. Moreover, the first preclinical study conducted in 1992 has already demonstrated the positive effect of mRNA application as a protein replacement. In the first study, a temporary reversal of diabetes insipidus (DI) took place in mice that were injected with the mRNA of vasopressin in the hypothalamus [41]. Interestingly, saRNA has also been suggested for preventing AD to raise ATP levels in the brain by reducing Aβ [9]. Notably, DM (both type 1 and type 2) remains an unsolvable global health disorder that still has insulin therapy as the main treatment. DM is an extremely common metabolic disorder that is characterized by abnormally elevated blood glucose levels. There are two main types of DM—type 1 DM (T1DM) and type 2 DM (T2DM). In T1DM, insulin is not produced by the pancreatic cells as the immune system destroys the insulin-producing β cells, while, in T2DM, either insulin secretion is impaired, or resistance to peripheral actions of insulin occurs and the body does not react to insulin [42]. The reasons for the development of T1DM include genes, and environmental factors like viruses while T2DM can be conditioned by obesity, physical inactivity, stress, etc. [43,44], and, as a result of T1DM and T2DM, hyperglycemia takes place. DM, on the other hand, increases the risk of many other diseases such as chronic kidney diseases, heart diseases, mental health, etc. [42,45]. Unlike DM, DI is a rare disease conditioned via the endocrine condition affecting approximately 0.004% of the world population. In this disease, the levels of the antidiuretic hormone vasopressin, which regulate the water and salts in the body, are downregulated and induce the consumption of extremely high amounts of water [46,47]. As the thirst is dramatically increased, the water intake reaches up to 20 L/day [48]. Currently, the only treatment is the use of the synthetic hormone desmopressin as a replacement for the deficient anti-diuretic hormone [48]. In order to overcome these health complications, the saRNA approach is hypothesized to replace insulin injections or induce vasopressin synthesis in the body by inserting the appropriate encoding gene into the saRNA construct and allowing the body to produce the necessary protein for long-lasting effects. Obviously, there will be difficulties to accomplish this hypothesis; however, this theory seems promising. An et al. have used mRNA-based technology with the delivery of biodegradable LNPs for protein replacement in mouse models of methylmalonic acidemia and demonstrated remarkably improved survival of the mouse models without manifesting liver toxicity [49]. Apart from that, AATD represents a hereditary disease that also might be a noteworthy target for saRNA-based therapeutics. AATD affects the liver and the lungs, and it is the main cause of chronic obstructive pulmonary disease (COPD) emphysema.

Besides the abovementioned, the saRNA approach might be considered for cosmetic uses such as alopecia treatment. In case of AGA, it has been proposed that miRNAs have the ability to inhibit certain proteins to activate signaling pathways involved in this process [14]. On the other side, the saRNA approach also has a huge potential to activate certain signaling pathways implicated in the hair growth process (such as Wnt/β-catenin) by an augmentation of certain peptides (Wnt ligand, β-catenin, etc.) into dermal papilla cells and initiate hair growth. Therefore, the current study suggests designing and developing saRNA approaches for protein replacement therapy that might become a new milestone for the treatment of numerous diseases.

All the abovementioned information supports the concept of saRNA application for protein replacement. In order to achieve the desired outcomes, the right strategy is the key. Here, we discuss the steps for saRNA research design in vivo. The first step is to design and synthesize the plasmid vector containing the GOI. The sequence of sgPr varies and depends on the selected alphavirus for the saRNA approach [50]. After the plasmid is transformed into the competent cells and amplified, the following step is to extract it and validate the expression of desired protein via in vitro analysis. In case the expression of the protein is satisfactory, the plasmid should be linearized and in vitro transcribed. Consequently, the obtained RNA construct will be packed with lipid nanoparticles (LNPs) [51] or other delivery formulations [52,53] and, eventually, administered to experimental animals with the appropriate administration route [54]. This is conditioned by the research purposes. For example, in case of immunization against the influenza virus, intradermal (I.D.), intramuscular (I.M.), or subcutaneous (S.C.) routes [55,56] can be used according to the designed experiment. Ultimately, the evaluation of in vivo analysis can be carried out. In vitro transcription (IVT) of the large-sized saRNA, however, is a challenge as typical IVT protocols are developed for smaller-size RNAs. IVT reaction comprises the following: linearized plasmid DNA with T7 promoter, nucleoside triphosphates (NTPs), ribonuclease inhibitor, magnesium ions as cofactor for T7 polymerase, pH buffer providing the optimal condition for the reaction. There are studies on making improvements in saRNA IVT. Samnuan et al. have demonstrated that several important reaction components can aid in obtaining a high yield of functional saRNA during the IVT. For example, saRNA with a size ranging from 1.8 to 12.4 kb can be produced via the properly adjusted balance of magnesium ions and NTPs, the use of acetate ions, etc. [57]. The line of studies demonstrates that the IVT of saRNA is performed successfully [58,59,60]. The capping can be performed during the IVT as well as post-transcriptionally. Remarkably, for saRNA capping, the use of the AU cap is suggested as it is advantageous compared to the AG cap, as the AU cap preserves the authentic alphavirus 5′ end, resulting in efficient capping and high yield [38].

**Figure 3:**
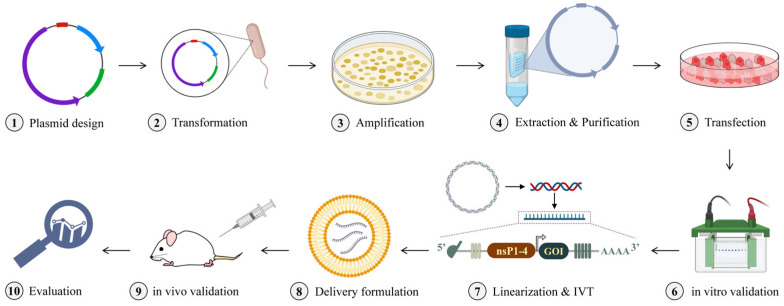
Schematic illustration of experimental design of saRNA treatment for protein deficiency disorders. nsP, non-structural protein; GOI, gene of interest; IVT, in vitro transcription.

The schematic illustration of the experimental workflow for the development of the saRNA approach for protein replacement therapeutic use is given in Figure 3.

## 6. saRNA for Single-Gene Disorders—Special Focus on AATD

Apart from its application as an immunization approach for infectious diseases, mRNA technology is thought to be a next-generation therapeutic strategy for single-gene disorders that are caused by the DNA change in a single gene [26]; hence, it is the fact that these disorders are inherited that is the reason for morbidity and premature mortality in families that are affected with these diseases [61]. Common single-gene disorders include hemophilia B—a bleeding disorder that is characterized by the deficiency of clotting factor IX, meaning that, in this condition, blood coagulation does not take place normally [62]. Another common single-gene disorder is a rare lipoprotein metabolism condition lecithin-cholesterol acyltransferase deficiency (LCATD) which results in severely reduced high-density lipoprotein (HDL) cholesterol and the clinical manifestation by corneal opacities, renal failure, and hemolytic anemia [26]. Interestingly, AATD also belongs to single-gene disorders and is a type of lung and liver disease conditioned by hereditary metabolic condition [63]. The mutation arises in the gene SERPINA1, which encodes the serine protease inhibitor (serpin)—AAT. As a result, the serum levels of AAT decrease well below the normal range. AAT is a protein that protects the lungs from the damage caused by inflammation that can lead to emphysema. AAT controls chemical reactions via inhibiting the activity of certain enzymes including neutrophil elastase (NE) in the lungs. In case of insufficient circulating levels of AAT, the levels of NE are drastically elevated, and this causes over-inhibition of a peptide called elastin—the main component of alveoli. Elastic tissue disruption is a major factor of pressure loss in alveoli and destruction of alveolar membranes, which results in the reduction of airflow and hence reduction of oxygen, further damage to the lungs, and, eventually, loss of function. Therefore, when the AAT is not going to the bloodstream and hence, in the lungs, circulating levels are decreased and the accumulation takes place within the liver where its synthesis occurs, lungs, as well as the liver become vulnerable. On the one hand, it cannot regulate the activity of elastase that makes NE free to attack the lungs and leads to emphysema, COPD, chronic bronchitis, etc., while on the other hand, it may lead to cirrhosis and increased risk of liver cancer. AATD is not a rare disease but is rarely diagnosed, although it can be simply and accurately detected via a blood test that measures the AAT levels. Notably, very often, underdiagnosis takes place as, when the patient has COPD or emphysema, AATD is often not considered while sometimes it is overdiagnosed [64]. Indeed, a misdiagnosis-related burden is a great challenge for understanding and managing the disease [65]. Often, many people who have AATD are misdiagnosed with asthma [66]. It happens as these two health conditions share the symptoms. In addition, people who have AATD respond well to asthma medicines. AATD was first described by Laurell and Erikson. Their case studies revealed the patients with very pronounced AATD, which was the new type of dysproteinemia in 1963 [67]. The healthy range of AAT serum levels is considered 20–53 µmol/L (100–220 mg/dL) [68], while people with serum AAT levels below 11 μmol/L (80 mg/dL) are at risk of developing COPD or liver diseases [26]. The main therapy for AATD is AAT pooled from the sera of the donors or the liver and lung transplantation that are very costly. Therefore, saRNA-based peptide replacement therapy is proposed for AATD. In addition, the preclinical study of modified systemic mRNA therapy for AATD already showed promising results [8].

## 7. saRNA for Cancer Immunotherapy

Recently, the development of cancer vaccines is the main focus of cancer research. Indeed, a number of methods are being developed to elicit a strong anti-tumor immune response. For designing the vaccine for cancer, the identification of tumor-associated antigens is of great importance. In addition, knowing the tumor microenvironment that allows the progression of the tumor and escaping from the host’s immune system is essential for the design of a cancer vaccine [69,70]. Cancer research focuses on several types of vaccines—antigen-based cancer vaccines that are based on tumor-associated proteins with different delivery systems [71]; peptide-based vaccines; cellular vaccines that are based on dendritic cells loaded with tumor antigens, allogenic tumor cell lines, autologous cancer cells; and nucleic acid vaccines including DNA and RNA-based approaches [72]. Considering its advantages including efficacy, safety, time, and cost-effectiveness, the mRNA vaccine strategy merits the most attention. mRNA vaccine against infectious diseases has already proved its benefits of application, while it is still in the clinical trials for cancer therapy [73]. On the other hand, saRNA technology has not been approved by the Food and Drug Administration (FDA) until now, but its promising results from clinical trials and superior characteristics compared with the conventional mRNA approach give rise to a new era of vaccinology [74]. Using the saRNA vaccine for cancer immunotherapy can achieve the robust expression of antigens in a short time. After the saRNA injection, desired candidate antigens are translated and presented via a major histocompatibility complex (MHC) I and II that induces a cellular and humoral immune response and results in tumor eradication [74]. Remarkably, there are a number of mRNAs in different phases of clinical development, e.g., mRNA-4157, mRNA-5671, and mRNA-4359 designed by Moderna as personalized cancer vaccine (PCV), Kirsten rat sarcoma (KRAS), and checkpoint vaccine, respectively [75]. According to the superiority of saRNA technique, basically, it can be used for every cancer vaccine development where a conventional mRNA approach is applied.

## 8. Delivery Systems for saRNA Therapeutics

The major challenge in developing saRNA vaccines is a delivery system that can carry saRNA to the target cells [4]. Moreover, the large structure of saRNA which can reach 15,000 nucleotides in length, makes cellular uptake more difficult. Until now, nanoparticles and nanoemulsions are used as delivery platforms in saRNA studies. These delivery platforms protect saRNA from degradation and facilitate uptake by the target cells [4]. There are degradable and non-degradable polymeric nanoparticles. For example, polyethylenimine (PEI), which is a cationic non-degradable polymer, has successfully delivered saRNA expressing influenza hemagglutinin of H1N1 A/Puerto Rico/8/1934 and A/California/7/2009 strains. Moreover, the saRNA-PEI induced remarkable protection in mice. A 64-fold less dose was enough to induce the same protection as non-replicating mRNA [6]. Apparently, the carrier systems that are applicable for mRNA or small interfering RNA (siRNA) delivery are not quite suitable for saRNA carriage as they are intended for the delivery of smaller size molecules—~2000–5000 nt (mRNA) and ~20 nt (siRNA), respectively. Blakney et al. have engineered a linear, cationic bioreducible polymer pABOL for influenza hemagglutinin saRNA delivery in mice. The study demonstrated that intramuscular and intradermal injection of saRNA with this delivery system resulted in enhanced protein expression. The transfection with a higher molecular weight of pABOL was efficient and protected the mice from challenge [76]. Another study has consolidated the abovementioned results in terms of high protein expression but also showed that LNP formulations are more immunogenic [77]. Indeed, LNPs are currently the most prevalent non-viral delivery for saRNAs as they require a minimal quantity of saRNA to evoke a strong immune response [78,79]. These systems have come to the fore of therapeutic platforms by pharmaceutical companies, especially since the successful development of SARS-CoV-2 mRNA LNP vaccines mRNA-1273 and BNT162b2 that are currently used worldwide [80,81].

The application of LNPs for the delivery of genetic materials originated from the development of liposomal drug carrier systems for small molecules. The principle of LNPs is to encapsulate the nucleic acid molecule into the system containing lipids that are organized in a bilayer form to deliver it into the target cells [21,82]. LNPs used for currently available COVID-19 vaccines contain four components with distinct functions: the ionizable lipids with the positive charge bind to the negatively charged backbone of the mRNA and enable RNA complexation; PEGylated lipids allow the stabilization and the longer systemic circulation of the particle via reducing antibody association and clearance by phagocytes; molecules of cholesterol and phospholipids contribute to the structure of the particle– allowing for packing the mRNA cargo into the LNPs. After the mRNA is packed in these components, it is protected from the destructive enzymes, transported, and successfully unloaded into the target cells to be translated into proteins [83]. These LNPs contain about 100 mRNA molecules per LNP [84] and are 80–100 nm in diameter [85,86]. Blakney et al. have formulated cationic LNPs and compared the LNP formulations with ionizable and cationic lipids with a diameter of 100–200 nm packing human immunodeficiency virus (HIV)-1 Env gp140 saRNA on the interior or adsorbed on the exterior of the particle. The results showed that both formulations induced similar antibody responses against the antigen. Moreover, LNPs containing cationic lipids protected saRNAs from nuclease degradation even when they were present on the surface [87]. These and the number of other preclinical [18,19,87,88] and clinical studies [12,78,89] demonstrate the feasibility and flexibility of LNPs for the saRNA approach [59,78,87,90,91,92,93,94,95,96,97]. On the other hand, the challenge of LNP formulations developed by Moderna and BioNTech/Pfizer is the requirement of the cold chain storage −20 °C and −70 °C, respectively. In response to this, Gerhardt et al. have studied the effectiveness of delivery of Zika saRNA with thermostable nonstructured lipid carrier (NLC). The RNA-NLC complex demonstrated stability for more than 8 months and more than 21 months at room temperature and 4 °C, respectively [98]. Alternatively, cationic nano-emulsions (CNEs) are water-in-oil emulsions that are also applicable for saRNA delivery [4,99,100,101,102,103]. They have already been demonstrated to elicit immune responses against influenza in mice and ferrets [100], HIV-1 in rhesus macaques [104], rabies in mice [105], etc. [4]. Besides the LNPs, polymeric nanoparticles, and nanoemulsions, adjuvanted saRNAs should also be considered. Manara et al. have studied the effect of saRNA encoding nucleoprotein of influenza A virus adjuvanted with a murine granulocyte–macrophage colony-stimulating factor (GM-CSF). The results demonstrated the induced nucleoprotein-specific immune response and enhanced recruitment of antigen-presenting cells (APCs) at the injection site [106]. Remarkably, immunization with naked saRNA has also been studied [107,108,109,110] and demonstrated efficiency against Zika virus, HIV-1, and influenza [4]. Electroporation has been successfully used to increase the delivery of saRNA after intramuscular administration with the broad immune response against HIV envelope protein in Balb/c mice [111] and moderate immune responses against Zika virus and protected the IFNAR1−/− mice from the viral challenge [110].

A number of other approaches for saRNA delivery have also been studied. These delivery systems include chitosan nanogel alginate (Chitosan NGA) [112], dendrimer [113], or modified dendrimer nanoparticle (MDNP) [113,114], cell-penetrating peptides (CPP) PEI [115], cationic lipids [116], polyplexes [117], natural lipopolyplexes [118], NLC [119], lipid inorganic nanoparticle emulsion (LION emulsion) [120], lipoplex [121], cationic adjuvant formulation (CAF) [122], virus-like particles [123], gene gun [92,124], and other techniques [40,125,126,127].

## 9. Challenges

**Figure 4:**
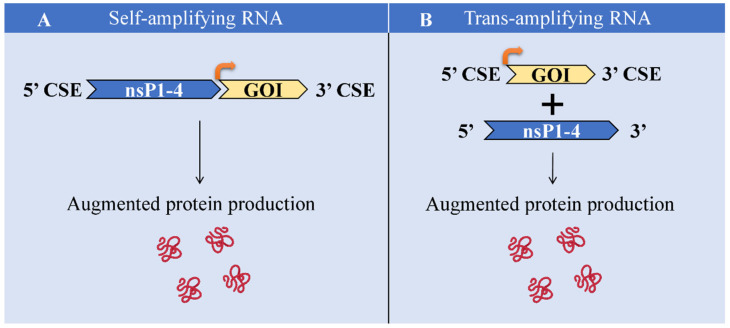
Comparison of saRNA and taRNA. (**A**) saRNA is a single large RNA molecule encoding nsP1-4 and GOI. As a result, protein of interest is produced in augmented levels. (**B**) taRNA represents two RNA molecules encoding GOI and nsP1-4 separately. As a result, protein of interest is produced in augmented levels. CSE, conserved sequence elements; nsP1-4, non-structural protein 1-4; GOI, gene of interest.

As for all of the novel approaches, the saRNA technique is also characterized by certain limitations that need to be overcome. The main obstacles are the lack of studies regarding the immunogenicity of RDRP complex, limited clinical data, the necessity of prime-boost administration [5], large size and complex sequence of the saRNA molecule [33], the challenge of efficient delivery, the larger size of the delivery system, shorter half-life [128], the proneness to degradation by nucleases [5], and induction of strong innate host immune responses [31,37]. There is no approved saRNA-based vaccine till now, which is also a burden. Nevertheless, advantages prevail over challenges. Remarkably, the challenge of the large size of saRNA can be partially overcome via splitting the whole sequence into nsPs and GOI in separate molecules and the application of trans-amplifying RNA (taRNA) [32,33]. taRNA has been demonstrated to be as effectively expressed as saRNA, pointing out the promising outcome of taRNA application [109]. The schematic illustration of saRNA and taRNA comparison is given in Figure 4.

## 10. Conclusions and Future Perspectives

The current review provides insights into the saRNA approach for protein replacement therapy. saRNA indeed has a huge potential to display a better RNA approach compared to a conventional mRNA technique, especially for those disorders which necessitate protein replacement therapy. Till now, all the clinical studies on saRNA have been performed in order to develop prevention strategies against infectious diseases (Table 1). This is mainly conditioned by the outbreak of COVID-19. Indeed, it is crucial for pandemics/epidemics preparedness to develop vaccines against the existing viruses to save lives. In the meantime, certain disorders that might be single-gene or non-heritable diseases that require protein replacement therapy should not be forgotten for the saRNA approach employment. Therefore, in terms of protein replacement therapy, saRNA certainly seems to exhibit distinctly advantageous benefits. For protein replacement purposes, the main advantage of the saRNA is its plausible long-lasting effects, the requirement of a much lower dosage that reduces the side effects markedly. Moreover, as the saRNAs allow for conferring immune response at low doses, a single-dose regimen might also be used in the future. However, the difficulties in this direction are noteworthy. The main challenge is the size of saRNA. The construct of saRNA appears to have a much larger size than conventional mRNA which also requires a suitable delivery vehicle for efficient uptake. In order to avoid this difficulty, taRNA is also proposed that represents two molecules instead of a single large-sized saRNA molecule. Nevertheless, there are substantially more data on saRNA itself compared to taRNA till now; thus, the latter approach requires more studies for further development. Fortunately, along with developing RNA technologies, carrier platforms also continue to advance, which results in the successful delivery of molecules as large as saRNA [129]. Evidently, the saRNA-based vaccine for COVID-19 (NCT05012943) is in clinical study Phase 3, and, therefore, it is close to getting approval and becoming the first approved saRNA-based vaccine. Here, we proposed the use of saRNA technique for protein replacement purposes for a number of diseases. Proteins including receptors and extracellular proteins can be generated via saRNA application without the risk of genome integration. This approach seems to have enormous potential for single-gene disorders, also for hereditary and some non-heritable diseases. As there is a vast number of pathologies in which certain genes are down-expressed and the normal function of the body is dysregulated, a new approach is emergent to overcome these huge obstacles. The proposed protein-replacing technique may be beneficial to AATD, DM, DI, or other health disorders. Furthermore, in order to exhibit satisfying results, studies on saRNA application for AD are also suggested [9]. Based on the promising results of mRNA for designing cancer vaccines, the saRNA approach seems even more promising owing to its superior characteristics.

There are crucial points to be addressed: determining the precise dosage of saRNA is essential. It is also important to evaluate the time period of the saRNA replication into the body. Time alike dosage might be dependent on the certainty of the disorder and the species of mammals. Multiple studies are required for this reason in the future.

Taken together, saRNA is gaining momentum worldwide due to the number of advantages that makes it superior to the conventional approaches [26]. On the other hand, looking back and going forward, saRNA can be used similarly but more efficiently than mRNA. A number of advantages of mRNA-based approaches [130] also indicate the promising future of saRNA-based protein replacement therapeutics. The simplicity, cost, and time-effective manufacturing technology of saRNA may allow the quick and successful development of protein replacement therapeutics.

## Figures and Tables

**Table 1:** Clinical trials that use mRNA technology for protein replacement in various diseases (accessed in September 2022). T2DM, type 2 diabetes mellitus; OTD, ornithine transcarbamylase deficiency.

Condition	ClinicalTrials.gov Identifier	Sponsor	Drug Name	Delivery Platform	Administration Route	Status	Completion Date
Heart failure	NCT03370887	AstraZeneca	AZD8601	Naked mRNA	Epicardial injection	Phase 2	30 June 2021
Ulcers associated with T2DM	NCT02935712	AstraZeneca	AZD8601	Naked mRNA	Intradermal	Phase 1	8 January 2018
Propionic acidemia	NCT04159103	ModernaTX, Inc.	mRNA-3927	In vivo/Lipid nano-systems	Intravenous	Phase 1 and 2	6 January 2027
Methylmalonic Acidemia	NCT03810690	ModernaTX, Inc.	mRNA-3704	In vivo/Lipid nano-systems	Intravenous	Withdrawn	18 August 2020
OTD	NCT03767270	Translate Bio, Inc.	MRT5201	In vivo/Lipid nano-systems	Intravenous	Withdrawn	July 2022
Cystic Fibrosis	NCT03375047	Translate Bio, Inc.	MRT5005	In vivo/Lipid nano-systems	Nebulization	Phase 1 and 2	December 2021

**Table 2:** List of the interventional clinical trials of self-amplifying RNA as a vaccine (accessed in September 2022). COVID-19, coronavirus disease 2019.

Condition	Location	Sponsors and Collaborators	Estimated Enrollment	Status	Start Date	NCT Number/Phase
COVID-19	Vietnam	Vinbiocare Biotechnology Joint Stock Company Arcturus Therapeutics, Inc.	19,400	Active, not recruiting	August 2021	NCT05012943 Phase 2/3
Influenza	United States	Pfizer	468	Recruiting	February 2022	NCT05227001 Phase 1
COVID-19	United States, Singapore	Arcturus Therapeutics, Inc.	72	Recruiting	September 2021	NCT05037097 Phase 1/2
COVID-19	Uganda	MRC/UVRI and LSHTM Uganda Research Unit	42	Recruiting	June 2021	NCT04934111 Phase 1
COVID-19	South Africa	ImmunityBio, Inc.	180	Recruiting	May 2022	NCT05370040 Phase 1/2
COVID-19	United States, Singapore	Arcturus Therapeutics, Inc.	600	Terminated	December 2020	NCT04668339 Phase 2
COVID-19	United States	HDT Bio Kaiser Permanente Rainier Clinical Research Center C3 Research Associates	63	Recruiting	November 2021	NCT05132907 Phase 1
COVID-19	United States	GlaxoSmithKline	10	Completed	February 2021	NCT04758962 Phase 1

## Data Availability

All data are available in the manuscript.
